# Evaluation of the Nutritional and Health Values of Selected Polish Mushrooms Considering Fatty Acid Profiles and Lipid Indices

**DOI:** 10.3390/molecules27196193

**Published:** 2022-09-21

**Authors:** Michalina Gałgowska, Renata Pietrzak-Fiećko

**Affiliations:** 1 Department of Meat Technology and Chemistry, Faculty of Food Sciences, University of Warmia and Mazury in Olsztyn, Cieszyński 1 Sq, 10-719 Olsztyn, Poland; 2 Department of Dairy Science and Quality Management, Faculty of Food Sciences, University of Warmia and Mazury in Olsztyn, Oczapowskiego 7 Str., 10-719 Olsztyn, Poland; 3 Department of Commodity Sciences and Food Analysis, Faculty of Food Sciences, University of Warmia and Mazury in Olsztyn, Cieszyński 1 Sq, 10-719 Olsztyn, Poland

**Keywords:** edible fungi, fatty acids, atherohenic index, thrombogenic index, hypocholesterolemic/hypercholesterolemic ratio, health-promoting index

## Abstract

*Imleria badia*, *Boletus edulis*, and *Cantharellus cibarius* are popular mushrooms of economic importance in Poland. Since physical and mental development of a person and the maintenance of good health entail providing the body with adequate nutrients, including plant and animal fats, the aim of this study was to determine the fatty acid profiles of three mushroom species from Poland and to assess their nutritional and health values using lipid indices. Studied mushrooms have a favorable fatty acid composition due to the high percentage of polyunsaturated fatty acids. Low values of the atherohenic index (AI) and the thrombogenic index (TI) prove that the consumption of the fungi may decrease the risk of coronary heart disease. Products with a high hypocholesterolemic/hypercholesterolemic ratio (H/H) and health-promoting index value are assumed to be more beneficial to human health, granting the possibility for using mushrooms in the nutrition of people with hypertension and in the prevention of cardiovascular diseases.

## 1. Introduction

Physical and mental development of a person and the maintenance of good health entail providing the body with adequate nutrients, including plant and animal fats. The fats in the human diet are generally fatty acids, which exist in nature in the form of mixtures of saturated (SFA), monounsaturated (MUFA), and polyunsaturated fatty acids (PUFA). Fatty acids are distributed to the cells, where they contribute to muscle contraction and regulate overall metabolism. Polyunsaturated fatty acids participate in many regulatory processes at the levels of cells, tissues, and the whole organism. The greatest interest is the signaling role of essential fatty acid (EFA) metabolites and their derivatives, especially those related to inflammatory processes. Deficiencies are manifested by disturbances in growth, functioning of the respiratory system, dermatoses, increased energy metabolism, and kidney damage [1]. However, works on the role of fat in maintaining human and animal health concern not only polyunsaturated but also monounsaturated and saturated fatty acids. Fatty acids can play a positive or negative role in the prevention and treatment of disease. The studies show that saturated fatty acids, especially palmitic (16:0), myristic (14:0), laurel (12:0), and to a lesser extent, stearic (18:0) acids, increase the concentration of cholesterol in low-density lipoproteins (LDL cholesterol). Saturated fatty acids can also be a factor in the development of certain cancers in both humans and other mammals. Some trans acids, formed as a result of hardening plant polyunsaturated fats, also have a similar unfavorable effect [2,3].

Both epidemiological and clinical studies have shown that fatty acids are associated with cardiovascular disease [4,5,6]. In addition, they also affect ailments such as: neurological diseases [7,8], non-alcoholic fatty liver disease [9], allergic disease [10], etc. Evidence indicates that the free FA profile is altered in both leukemia and pre-leukemia states, especially C14:0, C16:0, and C18:0 acids [11,12,13].

In general, the type of fat consumed should be taken into account when assessing the role of fatty acids in maintaining human and animal health. Fatty acids are obtained from various dietary sources that possess individual characteristics; therefore, fatty acid composition should be assessed. In addition, to determine the nutritional and medicinal value of different food products and raw materials, PUFA/SFA, atherohenic index (AI), thrombogenic index (TI), hypocholesterolemic/hypercholesterolemic ratio (HH), and health-promoting index (HPI) indices are used. They indicate the relationships between individual fatty acids or their groups.

Taking into account the various useful effects on health provided by fatty acids, the number of scientific studies on edible mushrooms, including their fatty acid composition, has increased with each passing day. Since ancient times, mushrooms have been considered valuable healthy foods. *Imleria badia*, *Boletus edulis*, and *Cantharellus cibarius* are popular mushrooms of economic importance in Poland. They are eagerly collected and consumed, but also, due to their beneficial physicochemical properties, they are used in trade, medicine, and pharmaceuticals.

*Boletus edulis*, also known as king bolete or porcini, is one of the most appreciated wild mushrooms. It comes from Europe, but it is distributed worldwide [14]. Its appearance is characteristic of the *Boletacea* family, which consists of a bun-shaped brown cap, a pleasant-smelling white flesh, and a white or light-brown stipe. This species is abundant in essential trace element such as selenium, zinc, and copper, as well as macronutrients such as potassium, magnesium, and calcium [15]. Compared to less common fungi, *B. edulis* demonstrates the highest antioxidant activity [16,17].

*Imleria badia*, called bay bolete in English, was previously described as *Imleria badia* (1832) and *Xerocomus badius* (1931). As with *B. edulis*, *I. badia* belongs to the Boletaceae family. This species grows next to tree trunks in coniferous and mixed forests in North America, Europe, Australia, and Japan as well as in China and the Southeast Asia region. *I. badia* is one of the most collected and valued edible mushrooms and is on the list of fungi approved for marketing and processing in European countries. It has an intense brown color of the hat, a firm flesh, and a pleasant aroma and taste. The fruiting body of *I. badia* has been shown to be a good source of proteins and free amino acids as well as carbohydrates and sugars. It mainly contains polyunsaturated fatty acids (PUFA) and other components with antioxidant activity (indole and phenolic compounds). The chemical composition of *I. badia* also includes proteins, vitamins, especially from the B group, as well as macro- and microelements [18].

*Cantharellus cibarius*, called gold chanterelles, belongs to the *Cantharellaceae* family. It is harvested almost all over the world and can be found in nature generally in the period from early autumn to early spring. The apricot-scented fruit of this mushroom and its alluring yellow color are highly appreciated throughout Europe. Numerous studies indicate that the fruiting body of *C. cibarius* is a great source of vitamins A, D, E, and C. Moreover, this species is also rich in ergocalciferol (vitamin D2). The fruiting body of *C. cibarius* is comprised of both saturated and unsaturated fatty acids, the content of which is in a proportion of 1:4. *C. cibarius* contains various trace elements, including zinc. Basing on numerous studies, it can be concluded that *C. cibarius* shows antioxidant, antimicrobial, anti-hypertensive, immunomodulatory, anti-inflammatory, and antiviral properties, among others [19,20].

Considering the above, the aim of this study was to determine the fatty acid profile of three mushrooms species from north-eastern Poland (three locations), as well as to assess their nutritional and health value with the use of lipid indices.

## 2. Results

In the experiment, 18 fatty acids were identified, from C12:0 to C20:2. The contents of C17:1, C22:1 n-11, and C22:1 n-9 acids in *C. cibarius* and the content of C18:1 cis-11 in *B. edulis* were not determined (Table 1). The percentage contents of saturated, monounsaturated, and polyunsaturated fatty acids in *I. badia*, *B.edulis* and *C. cibarius* from the studied regions are presented in Table 2 and Table 3).

The mean content of lipid substances in *I. badia*, *B. edulis*, and *C. cibarius* were 0.02%, 4.08%, and 2.39%, respectively. The results of the contents of saturated and unsaturated fatty acids in studied mushroom species are presented in Table 2. Among the saturated acids, C16:0 and C18:0 were dominant in most of the studied mushrooms. Only in the case of *C. cibarius* was the content of C20:0 was higher than that of C22:0.

Comparing the content of C16:0 acid between the studied regions, statistical differences were found only in B. edulis, where the highest value was determined for the B region (10.37%), the C region (9.03%), and then the A region (7.68%). The lowest content of C18:0 acid was found in I. badia from region B (1.82%). The values obtained for regions A and C did not differ statistically in this species and were equal to 2.53% and 2.33%, respectively. The content of this acid in the other species did not differ statistically between regions and exceeded 3%. However, significant differences were found in the content of C22:0 acid in *I. badia*. The highest content was found in region B (1.78%), followed by regions C (0.87%) and A (0.50%). In the case of B. edulis, statistically similar samples were from regions A (0.62%) and C (0.78%). About twice as much was specified in B. In the case of *C. cibarius*, no presence of this acid was found in region A, while in regions B and C, the contents were 0.68 and 0.54%, respectively. The content of C20:0 acid in the examined mushrooms did not differ significantly between regions. The sum of saturated fatty acids was statistically different in B. edulis, where it was 15.91% in region B, 13.87% in region C, and 12.37% in region A.

When analyzing the mean content of saturated fatty acids in individual species, significant differences were found between *C. cibarius*, *I. badia* and *B. edulis* in the case of C16:0 and C18:0. An amount of C16:0 acid almost two times lower than in *I. badia* (15.56%) was observed in *B. edulis* (9.21%), while in *C. cibarius*, 12.49% was determined. In the case of C18:0, the lowest value was found in *I. badia* (2.21%), and the highest value was found in *C. cibarius* (3.56%). The significantly lowest content of C22:0 acid was found in *C. cibarius* (Table 2).

Among monounsaturated fatty acids, C18:1 cis-9, C18:1 cis-11 (which was not found in *I. badia*) and, to a lesser extent, C16:1 dominated in most samples. The C18:1 cis-9 acid content did not differ between regions only in *C. cibarius* (A: 6.62%, B: 6.87%, C: 6.79%). In *I. badia*, the statistically highest value was determined in region A (43.74%), then in C: 37.66% and in B: 27.34%. In *B. edulis*, the following contents were observed: 42.25%, 31.65%, 25.55%, respectively. The content of C18:1 cis-11 acid in *C. cibarius* was the statistically highest in region A (21.65%). In the remaining regions, significantly lower values were determined: 19.98% for regions B and 19.85% for region C. The highest content of this acid in *B. edulis* was found in region B (6.80%). This value was statistically different from that obtained for region A (2.85%). In the case of C16:1 acid in the analyzed species of fungi, no significant differences in its content were found between the studied regions. Statistical differences between the regions were not observed in the sum of MUFA content in *C. cibarius* (A *=* 28.67%, B *=* 27.34%, and C *=* 27.13%). However, in the case of the other studied species, these values were significantly different (*B. edulis*: A *=* 46.87%, B *=* 33.48%, C *=* 37.77%; *I. badia*: A *=* 44.99%, B *=* 28.22%, C *=* 38.82%) (Table 2).

Differentiation in the mean content of C18:1 cis-9 acid was found between both *I. badia*, *B. edulis*, and *C. cibarius*, where the value was more than five times smaller than in the other species tested (6.76%). On the other hand, the content of the C18:1 cis-11 acid was over four times higher in *C. cibarius* (20.57%) than in *B. edulis*, while the presence of this acid was not found in *I. badia* (Table 2).

The dominant polyunsaturated acid among the mushroom species under study was C18:2 (Table 2). The content of this acid did not differ significantly between regions in *C. cibarius* (approx. 54%). Statistically significant differences were found in *I. badia* and the obtained values ranged from 35.31% in the A region to 50.81% in the B region. *B. edulis* was also characterized by a different content of this acid depending on the sampling site (A = 40.16%, B = 49.99%, C = 47.77%). The value of the sum of polyunsaturated fatty acids in *C. cibarius* was similar in all the examined regions (approx. 54%). In the case of other species, the highest levels were found in region B, then in C, the lowest in A (Table 2).

Analyzing the mean content of C18:2 between the studied species, no significant differences were found in the content of this acid between *I. badia* and *B. edulis*. In *C. cibarius*, about 10% more of this acid (53.94%) was found. Among the studied species of fungi, *C. cibarius* had the highest statistically significant share of polyunsaturated fatty acids (54.46%). In this species, 27.79% of monounsaturated acids and 17.75% of saturated acids were found. The significantly most saturated (19.80%) and the least polyunsaturated fatty acids (42.81%) were found in *I. badia*. Monounsaturated acids accounted for 40.28%. *B. edulis* contained the statistically lowest amount of saturated fatty acids (14.05%) and the highest amount of monounsaturated (40.28%). The share of polyunsaturated acids was 45.67% (Table 2).

Table 3 shows percentage ratio of SFA, MUFA, PUFA, UFA and the lipid quality indices calculated for studied mushrooms species. When analyzing the studied species of fungi, it was observed that *I. badia* had the highest values of MUFA/SFA (2.87), PUFA/SFA (3.25), UFA/SFA (6.12), H/H (8.42), and HPI (8.70). This species also had the lowest values of AI (0.12) and TI (0.28). In *I. badia*, the MUFA/SFA value did not differ statistically from the values obtained for other mushrooms. The inverse relationship was observed for PUFA/SFA and the HPI, where the values were significantly lower, and for the AI and TI, for which statistically higher values were observed. In *C. cibarius*, the value of PUFA/SFA was similar to that of *B. edulis*, while UFA/SFA was similar to that of *I. badia*. The values of AI and HPI did not differ significantly from those obtained for *B. edulis*. H/H was statistically similar to *I. badia*, while TI differed significantly from all studied species of fungi.

## 3. Discussion

Many reports have shown that edible mushrooms are characterized by a high percentage share of unsaturated fatty acids. In the current study, *C. cibarius* had the highest PUFA content among the studied species. The lowest SFA content was found for *B. edulis*. Heleno et al. [21], when examining mushrooms purchased in 2012 on the Polish market, determined approx. 3.5% less SFA in this species, while as much as 12% more PUFA. In the case of *I. badia*, the values determined by the authors were at a similar level. Different proportions were observed by Bengu [22] when researching *B. edulis* from Turkey. They determined more than twice the content of SFA and 15% less MUFA.

When comparing the studied mushrooms to raw materials and food products described in the literature, it can be concluded that the fatty acid profile of mushrooms is more similar to that of plant products than that of animal origin. This is reflected in a higher content of unsaturated acids than saturated acids. Lopes et al. [23], when examining grape flour, estimated the following amounts of acids: SFA, from 17.52 to 22.96%; MUFA, from 34.09 to 47.04%; and PUFA, from 30.00 to 48.39%. More than twice as much SFA (34.51–48.42%) and several times less PUFA (6.57–12.91%) were determined in the meat of pigs [24]. In hard cow, sheep, and goat cheeses, Paszczyk and Łuczyńska [25] determined SFA to be in the range of 54.60–59.41%; MUFA, 23.66–27.92%; and PUFA, 3.31–4.36%. Additionally, in fish, saturated fatty acids accounted for over 50% [26].

PUFA/SFA is the most commonly used index to evaluate the effects of diet on cardiovascular health (CVH). In creating the index, it was hypothesized that all PUFAs in the diet can reduce low-density lipoprotein (LDL-C) cholesterol and lower serum cholesterol, while all SFAs contribute to high serum cholesterol. Therefore, the higher this index, the more positive the effect. Among the examined mushrooms, *B. edulis* had the highest value of the index. It also showed a higher value of the MUFA/SFA ratio, and thus its UFA/SFA ratio was also the highest. The results obtained for mushrooms in this study are high when compared to those obtained by Nantapo et al. [27] for cow’s milk (0.02–0.04). The higher index value was obtained by Farajzadeh et al. [28], who analyzed sunflower oil (4.75–4.94).

Both atherogenic (AI) and thrombiogenic (TI) indices can be used to assess the potential effects of the fatty acid profile on cardiovascular health. Food products and raw materials with lower values of mentioned indices have better nutritional quality and their consumption may diminish the risk of coronary heart disease, but currently, there are no recommended levels for the AI and TI. The atherogenic index (AI) illustrates the relationship between the atherosclerotic saturated fatty acids (C12:0, C14:0, and C16:0, with the exception of C18:0) and the sum of the antiatherosclerotic unsaturated fatty acids [29,30]. This is why consuming foods with a lower AI can decrease the levels of total cholesterol and LDL-C in human plasma [31]. The AI value mostly ranges from 0 to 8.0; however, values from 4.0 to 5.0 are regarded as a generally accepted normal ceiling for a higher risk of cardiovascular disease [24]. The mushrooms studied in present work had a low AI value, and the lowest was observed for *B. edulis*. Analyzing this index, as in the case of PUFA/SFA, sunflower oil also shows a higher health quality (0.09–0.11) [32]. A much higher value of the index was determined for cow’s milk 4.08–5.13 [26], and fish were characterized by the following values: *Leiognathus bindus*, 1.476–1.493; *Upeneus sulphureus*, 1.308–1.445 [27].

To assess the degree of thrombogenicity in many fatty acid composition studies, the TI has been used. It indicates the relationship between the prothrombogenic fatty acids (C12:0, C14:0, and C16:0) and the antithrombogenic ones (MUFA, n-3, and n-6 PUFA); therefore, for cardiovascular health, the consumption of foods with a lower TI value is recommended [12,33]. Among the studied species of mushrooms, *B. edulis* had the lowest thrombogenic index. In the case of the next index, cow’s milk is the least favorable (3.22–5.04) [34] and the best is camelina oil (0.1) [35]. The TI in fish was at the following levels: *Leiognathus bindus*, 0.810–0.886; *Upeneus sulphureus*, 0.852–1.154 [27].

The hypocholesterolemic/hypercholesterolemic ratio (H/H) may more accurately reflect the effect of the fatty acid profile on cardiovascular disease than PUFA/SFA, as with the health-promoting index (HPI), proposed by Chen et al. [36]. The HPI is the inverse of the AI; therefore, the higher the values of both mentioned indices, the better nutritional quality of the studied products. While considering analyzed mushrooms, *B. edulis* was the species with the highest values of the indices. The studied mushrooms were characterized by much higher index values than cow’s milk (H/H, 0.34–0.75 [37]; HPI, 0.16–0.28 [38]). The high H/H value was determined by Ratusz et al. [35] for camelina oil (11.2–15).

**Figure 1:**
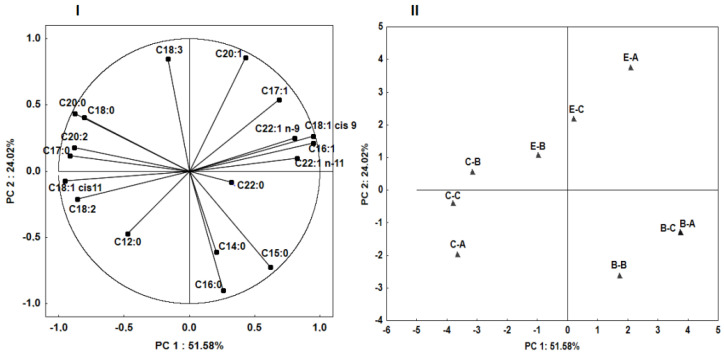
Principal component plot, variations in the parameters (fatty acids) of the analyzed mushrooms (**I**) score plot of the analyzed mushrooms (**II**). Abbreviations: first letters indicate mushroom species (B, *Imleria badia*; E, *Boletus edulis*; C, *Cantharellus cibarius*)*;* second letters indicate research region: A, B, C.

Principal component analysis (PCA) was performed on all samples and variables (analyzed fatty acids) to investigate the structure and regularity in the relationships between variables and cases. The first two principal components (PC) explained 75.6% of total data variance. The correlation between the original variables and the obtained principal components is shown in Figure 1 (I). Each of the variables are represented by the vectors. The direction and lengths of the vectors indicate to what extent the given variables affect the principal components. In the research, some of the variables (C20:0, C18:1 cis-11, C16:0, C15:0, C16:1, C18:1 cis-9, and C20:1) are located near the circle, i.e., the information contained in them is transferred by the principal component. Therefore, the information in the other input variables (C18:0, C20:2, C17:0, C18:2, C12:0, C14:0, C22:0, C22:1 n-11, C22:1 n-9, and C17:1) is transferred to a smaller degree by the principal components. Figure 1 (II) shows a plot of points in the plane of the principal components which illustrates the similarity between the fatty acid profiles of *Imleria badia, Boletus edulis*, and *Cantharellus cibarius*. The arrangement of the studied cases in relation to each other proves a difference in fatty acid profiles of the analyzed mushrooms in relation to mushroom species and location (region A, B, C). Additionally, it can be observed that within one species, the fungi of the *Boletus edulis* were the most diverse in terms of fatty acids.

## 4. Materials and Methods

### 4.1. Research Material

As part of the experiment, a total of 75 samples of *Imleria badia* (*n* = 25), *Boletus edulis* (*n* = 25), and *Cantharellus cibarius* (*n* = 25) were analyzed in terms of determining the profile of fatty acids and calculating health lipid indices. The samples were obtained in three different regions of Warmia and Mazury district (north-east Poland) differing in the degree of industrialization, the number of inhabitants, and the area of green areas (A, B, C). “A” is a city with a population of over 170,000, “B” has a population below 20,000 and “C” has a population over 30,000. Mushrooms were bought in purchase centers as a fresh material. Next, in laboratory conditions, they were dried and stored under appropriate conditions for further analysis. Hereinafter, the species of mushrooms will be called *I. badia*, *B. edulis*, and *C. cibarius*.

### 4.2. Analytical Methods

#### 4.2.1. Lipid Extraction and Determination

The homogenized material (the dried samples—approximately 10 g per sample— were ground to a powder with a laboratory homogenizer) was cold extracted with a mixture of dichloromethane and methanol (2:1, *v*/*v*) [39]. The samples were then filtered and transferred to a new tube to which 50 mL of an aqueous KCl solution (0.88%, *w*/*v*) was added. After that, the samples were centrifuged at 1500 rpm and 4 °C for 5 min. The lower layer (organic phase), after separation, was evaporated at 60 °C on a rotary evaporator.

At the same time, the representative samples of fungi included in the experiment were subjected to lipid extraction by the Soxhlet method, where the organic solvent was petroleum ether. The obtained mixture was subjected to evaporation in a vacuum evaporator, and the separated lipid substances were dried in an electric dryer at a temperature of 60 °C until constant weight was obtained. On the basis of the weight of the obtained lipids, the percentage of fat in the mushrooms was determined.

#### 4.2.2. Fatty Acid Profile Determination

The obtained lipids were subjected to esterification in order to determine the fatty acid composition. The derivatization of fatty acids to methyl esters was carried out in accordance with EN ISO 12966-1:2014/AC:2015 [40]. A total of 100 mg of lipids were transferred to a glass tube, and 4 mL of 2 M NaOH was added and heated on a heating block. After 10 min, 5 mL of BF3 complex was added with continued heating. Then, 3 mL of isooctane was added to the boiling mixture and heated for 1 min. After removing the tube from the heat source, without cooling it down, 20 mL of 1% NaCl solution was added. Finally, 2 mL of the upper isooctane layer was transferred to a vial and subjected to analysis.

#### 4.2.3. Fatty Acid Chromatographic Separation and Identification

The chromatographic separation of the fatty acid esters was carried out using a gas chromatograph 7890 A Agilent Technologies (Agilent Technologies, Inc., Santa Clara, CA, USA) with a flame ionization detector (FID), capillary column Supelcowax 10 (length = 30 m, inside diameter = 0.32 mm, liquid phase = Supelcowax 10, film thickness = 0.25 μm); temperature: detector = 250 °C, dispenser = 230 °C, and column = 195 °C; carrier gas helium, flow rate = 1.5 mL/min (51 cm/s), split 50:1. The identification of fatty acids was performed on the basis of their retention time in relation to the retention time of standards of fatty acid methyl esters. For this purpose, a mixture of 37 standards (Supelco™ 37 Component FAME Mix, Supelco, Bellefonte, PA, USA, 10 mg/mL in methylene chloride (varied)) was applied. For the calculation of the percentage share of fatty acids, the computer program ChemStation was used.

### 4.3. Lipid Quality Indices

The lipid quality indices were calculated according to the fatty acid profile with use of the following formulae:

#### 4.3.1. Atherogenic Index (AI) Developed by Ulbritcht and Southgate [33]


(1) $$AI=\frac{C12:0+\left(4\times C14:0\right)+C16:0}{n-3\;PUFA+n-6\;PUFA+MUFA}\;$$

#### 4.3.2. Thrombogenic Index (TI) Developed by Ulbritcht and Southgate [33]


(2) $$TI=\frac{C14:0+C16:0+C18:0}{0.5\times \;\Sigma MUFA+0.5\times n-6\;PUFA+3\times n-3\;PUFA+\frac{n-3\;PUFA}{n-6\;PUFA}}\;$$

#### 4.3.3. Hypocholesterolaemic/Hypercholesterolaemic Ratio (HH) Developed by Santos-Silva and Santos-Silva [41]


(3) $$H/H=\frac{cis\;C18:1+\Sigma PUFA}{C12:0+C14:0+C16:0}$$

#### 4.3.4. Health-Promoting Index (HPI) Developed by Chen et al. [36]


(4) $$HPI=\frac{\Sigma UFA}{C12:0+4\times C14:0+C16:0}$$

### 4.4. Statistical Methods

Statistical analyses, including calculation of the mean value and the standard deviations, were performed with Microsoft Excel software (Microsoft 365). The significance of difference of the mean values between the samples was determined with the program Statistica 12 (Statistica 12, StatSoft Inc., Tulsa, OK, USA). Normality of the data distribution was analyzed using the Shapiro–Wilk’s test, whereas variance homogeneity was verified using Levene’s test. Non-parametric tests (Mann–Whitney U and Kruskal–Wallis) were used to compare the mean values of the features between the samples due to the non-fulfillment of the requirements for normal distribution of data and variance homogeneity. Using both tests, the significance level was 0.05. The assumed grouping factors were the species of mushrooms, as well as the place of their collection. Principal component analysis (PCA) was carried out to show the differences between the studied mushroom species and regions. The results of the experiment and their statistical interpretation are presented in the table and the figure.

## 5. Conclusions

In conclusion, the studied species of mushrooms have a favorable composition of fatty acids due to the high percentage of unsaturated, mainly polyunsaturated, fatty acids. They contain linoleic (C18:2) and linolenic (C18:3) acids, belonging to the families of n-6 and n-3 acids, which are important from a health point of view. Low values of the AI and TI prove that the consumption of the studied fungi may reduce the levels of total cholesterol and LDL-C in human blood plasma and decrease the risk of coronary heart disease. Additionally, products with a high H/H ratio and HPI value are assumed to be more beneficial to human health. When comparing the obtained indices with others of plant and animal products, the fungi were closer to the plant value ranges. They are characterized by satisfactory values of indices, which proves their pro-health properties. This is of importance to diets, due to the possibility of using mushrooms in the nutrition of people such as those with hypertension and in the prevention of cardiovascular diseases.

## Figures and Tables

**Table 1:** The presence of fatty acids in the lipids separated by mushrooms under study.

FA		Common/IUPAC Name	*Imleria* *badia*	*Boletus* *edulis*	*Cantharellus cibarius*
**SFA**	C12:0	lauric acid/dodecanoic acid	+	+	+
	C14:0	myristic acid/tetradecanoic acid	+	+	+
	C15:0	pentadecanoic	+	+	+
	C16:0	palmitic acid/hexadecanoic acid	+	+	+
	C17:0	margaric acid/[heptadecanoic acid	+	+	+
	C18:0	stearic acid/octadecanoic acid	+	+	+
	C20:0	arachidic acid/icosanoic acid	+	+	+
	C22:0	behenic acid/docosanoic acid	+	+	+
**MUFA**	C16:1	palmitoleic acid/(Z)-hexadec-9-enoic acid	+	+	+
	C17:1	heptadecenoic acid/(Z)-heptadec-10-enoic acid	+	+	nd
	C18:1 cis-9	oleic acid/(Z)-octadec-9-enoic acid	+	+	+
	C18:1 cis-11	vaccenic acid/(E)-octadec-11-enoic acid	+	nd	+
	C20:1	gadoleic acid/(Z)-icos-9-enoic acid	+	+	+
	C22:1 n-11	cetoleic acid/(Z)-docos-11-enoic acid	+	+	nd
	C22:1 n-9	erucic acid/(Z)-docos-13-enoic acid	+	+	nd
**PUFA**	C18:2	linoleic acid/(9Z,12Z)-octadeca-9,12-dienoic acid	+	+	+
	C18:3	linolenic acid/(9Z,12Z,15Z)-octadeca-9,12,15-trienoic acid	+	+	+
	C20:2	8Z,11Z-eicosadienoic acid/(8Z,11Z)-icosa-8,11-dienoic acid	+	+	+

**Table 2:** Percentage content of saturated (SFA), monounsaturated (MUFA), and polyunsaturated (PUFA) fatty acids in studied mushrooms from different regions of Poland.

FA	*Imleria badia*				*Boletus edulis*				*Cantharellus cibarius*			
	Regions				Regions				Regions			
	A	B	C	Mean	A	B	C	Mean	A	B	C	Mean
C12:0	0.09 ± 0.03 a	0.07 ± 0.02 b	0.08 ± 0.02 a	0.08 ± 0.02 b	0.06 ± 0.01 b	0.07 ± 0.01 a	0.07 ± 0.02 ab	0.06 ± 0.03 b	0.21 ± 0.15 a	0.07 ± 0.10 a	0.10 ± 0.0 a	0.12 ± 0.08 a
C14:0	0.18 ± 0.02 a	0.16 ± 0.003 a	0.17 ± 0.02 a	0.17 ± 0.01 a	0.15 ± 0.03 a	0.16 ± 0.04 a	0.15 ± 0.04 a	0.15 ± 0.03 a	0.19 ± 0.01 a	0.14 ± 0.03 b	0.15 ± 0.03 b	0.16 ± 0.03 a
C15:0	0.49 ± 0.05 b	0.69 ± 0.07 a	0.55 ± 0.11 b	0.58 ± 0.11 a	0.09 ± 0.003 c	0.17 ± 0.03 a	0.13 ± 0.05 b	0.13 ± 0.04 b	0.17 ± 0.02 a	0.17 ± 0.03 a	0.18 ± 0.03 a	0.17 ± 0.03 b
C16:0	15.29 ± 0.67 a	15.81 ± 0.59 a	15.28 ± 0.97 a	15.56 ± 0.72 a	7.68 ± 0.24 c	10.37 ± 0.53 a	9.03 ± 1.47 b	9.21 ± 1.11 c	12.14 ± 1.13 a	12.63 ± 2.00 a	12.69 ± 1.27 a	12.49 ± 1.53 b
C17:0	0.07 ± 0.01 b	0.10 ± 0.01 a	0.08 ± 0.02 b	0.09 ± 0.02 c	0.092 ± 0.00 b	0.12 ± 0.02 a	0.10 ± 0.02 b	0.11 ± 0.02 b	0.11 ± 0.02 a	0.13 ± 0.03 a	0.13 ± 0.02 a	0.12 ± 0.02 a
C18:0	2.53 ± 0.22 a	1.82 ± 0.14 b	2.33 ± 0.31 a	2.21 ± 0.39 c	3.13 ± 0.05 a	3.00 ± 0.39 a	3.03 ± 0.33 a	3.06 ± 0.27 b	3.53 ± 0.15 a	3.56 ± 0.25 a	3.61 ± 0.13 a	3.56 ± 0.19 a
C20:0	0.32 ± 0.05 a	0.27 ± 0.07 a	0.31 ± 0.04 a	0.30 ± 0.06 c	0.55 ± 0.02 a	0.60 ± 0.09 a	0.58 ± 0.06 a	0.57 ± 0.06 b	0.62 ± 0.11 a	0.71 ± 0.32 a	0.77 ± 0.31 a	0.70 ± 0.27 a
C22:0	0.50 ± 0.13 c	1.78 ± 0.60 a	0.87 ± 0.83 b	1.08 ± 0.78 a	0.62 ± 0.21 b	1.43 ± 0.75 a	0.78 ± 0.41 b	0.95 ± 0.60 a	0.00 ± 0.00 b	0.68 ± 0.48 a	0.54 ± 0.37 a	0.42 ± 0.46 b
C16:1	0.83 ± 0.05 a	0.74 ± 0.06 a	0.79 ± 0.09 a	0.79 ± 0.07 a	0.82 ± 0.03 a	0.66 ± 0.22 a	0.75 ± 0.019 a	0.74 ± 0.17 a	0.39 ± 0.02 a	0.38 ± 0.07 a	0.40 ± 0.07 a	0.39 ± 0.06 b
C17:1	0.03 ± 0.03 a	0.00 ± 0.00 a	0.03 ± 0.04 a	0.02 ± 0.01 a	0.06 ± 0.01 a	0.00 ± 0.00 c	0.02 ± 0.03 b	0.03 ± 0.03 a	0.00 ± 0.00 a	0.00 ± 0.00 a	0.00 ± 0.00 a	0.00 ± 0.00 b
C18:1 cis-9	43.74 ± 3.01 a	27.34 ± 2.16 c	37.66 ± 9.30 b	36.31 ± 8.74 a	42.25 ± 0.42 a	25.55 ± 2.10 c	31.65 ± 8.38 b	34.20 ± 8.52 a	6.62 ± 0.53 a	6.87 ± 0.65 a	6.79 ± 0.64 a	6.76 ± 0.60 b
C18:1 cis-11	0.00 ± 0.00 a	0.00 ± 0.00 a	0.00 ± 0.00 a	0.00 ± 0.00 c	2.85 ± 0.07 b	6.80 ± 3.15 a	4.71 ± 3.34 ab	4.72 ± 2.95 b	21.65 ± 1.98 a	19.98 ± 1.56b	19.85 ± 0.70 b	20.57 ± 1.69 a
C20:1	0.28 ± 0.03 a	0.14 ± 0.01 c	0.21 ± 0.07 b	0.21 ± 0.07 b	0.79 ± 0.02 a	0.47 ± 0.02 c	0.59 ± 0.16 b	0.63 ± 0.16 a	0.00 ± 0.00 b	0.10 ± 0.06 a	0.08 ± 0.08 a	0.06 ± 0.03 c
C22:1 n-11	0.06 ± 0.10 b	0.00 ± 0.00 c	0.08 ± 0.10 a	0.04 ± 0.02 a	0.05 ± 0.05 a	0.00 ± 0.00 c	0.02 ± 0.00 b	0.03 ± 0.05 a	0.00 ± 0.00 a	0.00 ± 0.00 a	0.00 ± 0.00 a	0.00 ± 0.00 b
C22:1 n-9	0.04 ± 0.09 a	0.00 ± 0.00 b	0.06 ± 0.02 a	0.03 ± 0.01 a	0.05 ± 0.04 a	0.00 ± 0.00 c	0.02 ± 0.03 b	0.03 ± 0.04 a	0.00 ± 0.00 a	0.00 ± 0.00 a	0.00 ± 0.00 a	0.00 ± 0.00 b
C18:2	35.31 ± 3.54 c	50.81 ± 4.08 a	41.24 ± 9.06 b	42.25 ± 8.14 b	40.16 ± 0.51 c	49.99 ± 1.48 a	47.77 ± 5.19 b	44.80 ± 5.04 b	53.87 ± 2.99 a	54.03 ± 1.68 a	54.18 ± 2.53 a	53.94 ± 2.30 a
C18:3	0.07 ± 0.02 c	0.134 ± 0.02 a	0.10 ± 0.04 b	0.10 ± 0.04 b	0.34 ± 0.01 a	0.33 ± 0.15 a	0.33 ± 0.11 a	0.33 ± 0.10 a	0.10 ± 0.02 b	0.13 ± 0.03 a	0.11 ± 0.02 ab	0.11 ± 0.03 b
C20:2	0.17 ± 0.03 a	0.14 ± 0.03 b	0.16 ± 0.03 a	0.15 ± 0.03 c	0.26 ± 0.004 b	0.28 ± 0.002 a	0.27 ± 0.02 ab	0.27 ± 0.02 b	0.397 ± 0.05 a	0.41 ± 0.03 a	0.41 ± 0.06 a	0.40 ± 0.12 a
Ʃ SFA	19.47 ± 0.77 a	20.69 ± 1.20 a	19.68 ± 1.43 a	19.80 ± 1.20 a	12.37 ± 0.24 c	15.91 ± 0.82 a	13.87 ± 1.81 b	14.05 ± 1.81 c	16.97 ± 1.34 a	18.09 ± 2.72 a	18.17 ± 1.82 a	17.75 ± 2.11 b
ƩMUFA	44.99 ± 3.10 a	28.22 ± 1.27 c	38.82 ± 8.89 b	37.40 ± 5.19 a	46.87 ± 0.44 a	33.48 ± 0.98 c	37.77 ± 5.41 b	40.28 ± 4.88 a	28.67 ± 2.52 a	27.34 ± 1.87 a	27.13 ± 0.69 a	27.79 ± 1.91 b
Ʃ PUFA	35.55 ± 3.56 c	51.09 ± 4.08 a	41.51 ± 9.08 b	42.81 ± 5.16 b	40.76 ± 0.51 c	50.60 ± 1.57 a	48.37 ± 5.20 b	45.67 ± 5.91 b	54.37 ± 2.96 a	54.57 ± 1.70 a	54.71 ± 2.50 a	54.46 ± 2.28 a

Values with the same letters do not differ significantly among the species between the regions at the significance level *p* < 0.05.

**Table 3:** Percentage ratio of SFA, MUFA, PUFA, UFA and the lipid quality indices calculated for studied mushrooms species.

	MUFA/SFA	PUFA/SFA	UFA/SFA	AI	TI	H/H	HPI
*I. badia*	1.89 ± 0.45 ab	2.16 ± 0.41 b	4.05 ±0.30 b	0.21 ± 0.01 a	0.45 ± 0.02 a	4.97 ± 0.33 b	4.90 ± 0.30 b
*B. edulis*	2.87 ± 0.83 a	3.25 ± 0.17 a	6.12 ± 0.88 a	0.12 ± 0.02 b	0.28 ± 0.04 c	8.42 ± 1.66 a	8.70 ± 1.49 a
*C. cibarius*	1.57 ± 0.25 b	3.07 ± 0.46 a	4.63 ± 0.67 b	0.16 ± 0.02 b	0.39 ± 0.05 b	4.76 ± 0.74 b	6.21 ± 0.93 a

Values with the same letters do not differ significantly between species at the significance level *p* < 0.05.

## Data Availability

Not applicable.
